# Public perception of the lifetime morbid risk of mental disorders in the United States and associations with public stigma

**DOI:** 10.1186/s40064-016-2974-y

**Published:** 2016-08-12

**Authors:** Nicholas D. Lawson

**Affiliations:** 151 Charles St, New York, NY USA

**Keywords:** Mental illness, Mental disorder, Prevalence, Social distance, Stigma, Lifetime morbid risk, Mechanical Turk, Online

## Abstract

**Purpose:**

This study examined the relationship between estimates of the prevalence of mental disorders and mental health stigma. It also examined whether stigma might be more greatly associated with the terms “mental illness,” “mental disorder,” or “mental health condition.”

**Methods:**

Respondents (*N* = 302) on Amazon’s Mechanical Turk completed an online survey designed to measure social distance, which is one variant of stigma. Half of the respondents were informed at the beginning of the survey that the lifetime morbid risk (LMR) of meeting criteria for at least one mental disorder at some point in life was 70–80 %, while the others were asked to provide their own LMR estimates. All respondents were also randomly assigned to view the survey with either the term “mental illness,” “mental disorder,” or “mental health condition.”

**Results:**

Higher LMR estimates (B = −0.030; β = −0.154), having a mental disorder (B = −2.002; β = −0.200), and a history of contact with an individual with a mental disorder (B = −2.812; β = −0.298), each significantly predicted lower desire for social distance. Respondents in the “mental disorder” group endorsed greater desire for social distance. Participants who were informed about LMR at the start of the survey did not score lower on social distance.

**Conclusions:**

Estimates for LMR were more than half as predictive of social distance scores as contact with individuals with mental disorders. But anti-stigma interventions may need to do more than inform individuals about the high prevalence of mental disorders in order to be effective.

**Electronic supplementary material:**

The online version of this article (doi:10.1186/s40064-016-2974-y) contains supplementary material, which is available to authorized users.

## Background

### Mental health stigma

The majority of Americans will meet criteria for at least one mental disorder by age 29 (Kessler and Wang 2008). Yet the stigma associated with these conditions is considerable, and may contribute even greater to the morbidity of a condition than the disorder itself (Hinshaw and Stier 2008). Global agencies, such as the World Health Organization and World Psychiatric Organization, national programs, and other leading authorities have identified stigma as the biggest problem facing psychiatry today (Sartorius 2004; World Health Organization 2001; World Psychiatric Association 2016; Young Minds 2010).

In surveys, US employers express more negative attitudes about hiring workers with psychiatric disabilities than almost any other group (Cook 2006), and 43.8 % are uncomfortable hiring those in treatment for depression (Scheid 1999). Worldwide, the public stigma of mental disorders is widely prevalent, even among mental health professionals (Nordt et al. 2006; Schulze 2007), and components of stigma have now been linked empirically with treatment avoidance (Clement et al. 2015; Mojtabai 2010).

The stigma of mental disorders is somewhat difficult to understand in the face of evidence that suggests their very high prevalence in the general population. By age 29, lifetime prevalence for having any mental disorder is 58.7 % by retrospective studies (National Comorbidity Survey Replication 2007), but prospective studies have found prevalence estimates to be double those of retrospective studies for individual disorders (Moffitt et al. 2010; Takayanagi et al. 2014). Lifetime morbid risk (LMR), or the probability of meeting criteria for a mental disorder at any point in one’s life from birth to death, should be even higher, but there are currently no known estimates or relevant data.

### Stigma and prevalence estimates

Stigma, as conceived by many theorists, provides a majority group with system-justification for discriminatory behavior against a minority (Corrigan et al. 2003; Jones and Corrigan 2014; Link and Phelan 2001; Nosek et al. 2009). Crucial to this end is the formation of stereotypes about the minority to establish its difference as the “other” group (Corrigan et al. 2003). If all, or nearly all, Americans are believed to be likely to have a “mental disorder” at some point in their lives, it should, presumably, be more difficult to sustain such perceived differences. Conceivably, the persistence of stigma might be related to a lack of public knowledge about the very high prevalence of mental disorders.

Only one previous study, Von dem Knesebeck et al. (2013), appears to have examined such a relationship. The study measured social distance, which is one particular variant of stigma. Respondents were contacted over the phone and provided vignettes describing individuals with the specific disorders of either depression, schizophrenia, anorexia nervosa, or bulimia nervosa, followed by the diagnosis. The participants were asked to estimate the LMR of the condition and to complete a survey designed to assess their desire for social distance from individuals such as those described in the vignette. The authors found that higher estimates for schizophrenia were associated with less desire for social distance, but there were no significant associations for the other disorders. A 1 % increase in estimated LMR for schizophrenia was associated with a 0.04 point decrease in scores for desired social distance (Von dem Knesebeck et al. 2013).

Yet estimates of the LMRs for individual disorders may not be related to LMR estimates for having any disorder. Some studies, including Von dem Knesebeck et al. have found that the public tends to overestimate the prevalence for specific disorders (Economou et al. 2009; Mond et al. 2004), but underestimate the prevalence for having at least one mental disorder of any type (Bourget et al. 2007; Department of Health 2003; Kemali et al. 1989). Von dem Knesebeck et al. (2013) did not find a relationship between social distance and LMR estimates for specific disorders, but the association between social distance and LMR estimates for all mental disorders has never been studied.

### Terminology

At the time this study was designed, it was not clear which particular term should be used in the surveys that would be completed by participants. Terms such as “mental illness,” “mental disorder,” and “mental health condition” are used interchangeably in the *Diagnostic and Statistical Manual of Mental Disorders, fifth edition* (American Psychiatric Association 2013), and Szeto et al. (2013) found that such terms were associated with similar levels of stigma. But there are still many reasons to suspect that the public might view the terms differently. For example, “mental illness” suggests severity (Szeto et al. 2013), while “mental disorder” implies a difference from normal. If social distance depends primarily on conceptualizations of the stigmatized as different (Corrigan et al. 2015), then perhaps the term “mental disorder” would be most likely to evoke the respondents’ desire for social distance. In spite of the findings from previous research, it was hypothesized that these 3 terms would significantly differ on associated social distance when measured here in this study. The term “mental disorder” will be used for the remainder of this article, unless otherwise noted.

The main hypothesis of this study was based on the presumption that respondents believing that a higher percentage of the population will meet criteria for a mental disorder at some point in life should also hold less stigmatizing views about the disorders than those who believe they are less common. Specifically, it was expected that high LMR estimates would be negatively associated with scores for social distance. It was also expected that providing information about the very high prevalence of mental disorders at the start of the survey would lead respondents to endorse less stigmatizing views of those with mental disorders.

## Methods

### Sample

The study was conducted through an anonymous online survey, given reports that online self-complete surveys, in contrast with face-to-face assessments, are relatively free of social desirability bias when measuring behavioral intentions toward people with mental disorders (Henderson et al. 2012). Respondents were recruited and surveys administered through Amazon’s Mechanical Turk (MTurk), which has been found to yield data at least as reliable and valid as that obtained through methods traditionally employed in psychology and the social sciences (Buhrmester et al. 2011; Paolacci and Chandler 2014; Shapiro et al. 2013). MTurk samples include only those who are 18 years or older, have demographics that generally resemble the US, though with some differences, and model a random population (Paolacci and Chandler 2014; Paolacci et al. 2010; Shapiro et al. 2013).

Three hundred participants were recruited. A notice was posted on the MTurk Human Intelligence Tasks list requesting workers to “answer a survey about mental health” that would take no more than 15 min to complete. All tasks were completed within hours of the posted notice. Workers on MTurk were only eligible to participate if they resided in the US, had completed at least 50 prior assignments on MTurk, and had a prior task approval rating of at least 95 %. These requirements are standard for research with MTurk populations and ensured high quality data by excluding individuals who might end the survey midway and thereby bias the results. Participants who successfully completed the survey were paid $0.40. In general, however, MTurk workers are internally motivated and will readily participate in research of this kind for much less than this amount (Buhrmester et al. 2011). The study was determined to be exempt from review by an Institutional Review Board.

### Demographics

Respondents answered a number of items on demographics. For contact, they were asked if they had ever known someone with a mental disorder. For personal experience, respondents were asked, “Do you have a mental disorder?” and affirmative responses were considered to indicate personal experience with a mental disorder. The answers were based on the perception of the informant, the same approach taken by Von dem Knesebeck et al. (2013). In other studies, however, only those told they had a mental health diagnosis by a mental health provider were considered to have a disorder, and some studies also required a history of treatment. Yet 67 % of those with mental disorders do not receive treatment (Kessler et al. 2005), and the approach taken here therefore seemed more likely to capture those with the variable of interest.

Table 1 summarizes the characteristics of the overall sample, which were consistent with the typical demographic profiles of MTurk. Participants ranged in age from 18 to 75. Compared with the general US population, the respondents in this study were younger, with a higher proportion of males and whites, more highly educated, and also more likely to be unemployed. The mean of respondents’ estimates for the LMR of having any mental disorder was 32.95 (SD = 19.89).

**Table 1:** Demographics of all respondents (*N* = 302)

	Mean (SD) or frequencies
Age	34.4 (11.8)
Gender, female	42.1 %
Race	
White	75.8 %
Hispanic	4.6
Non-Hispanic black	8.6
Other	10.9
Highest educational attainment	
Bachelor’s degree or higher	46.7 %
At least 1 year college	35.4
12th grade high school	13.9
11th grade high school	2.0
Employment	
Employed full-time	51.7 %
Employed part-time	15.9
Unemployed	26.2
Full-time student	6.3
Personal experience, yes	23.5 %
Contact, any	79.1 %

### Measures

To determine if a particular term was more associated with desire for social distance, participants were randomly assigned to view the survey with either the term “mental illness,” “mental disorder,” or “mental health condition.” Half the participants (*n* = 151) were informed at the beginning of the survey that the LMR of having at least one mental disorder for an individual in the US is 70–80 %, in order to see if this had any effect on stigma scores. The other half (*n* = 151) were not provided with this information, and they were asked, instead, to provide their own estimates. They were asked, “What percentage of the current US population has, has had, or will have a mental disorder in their lifetime?” This resulted in 6 distinct groups, as seen in Table 2.

**Table 2:** Mean (and standard deviation) social distance scores by terminology group among those informed and those not informed of prevalence estimates

	“Mental illness”		“Mental disorder”		“Mental health condition”		All	
	Mean (SD)	*n*	Mean (SD)	*n*	Mean (SD)	*n*	Mean (SD)	*n*
Informed	12.28 (3.94)	47	12.34 (4.61)	50	11.31 (3.88)	54	11.95 (4.16)	151
Not informed	11.32 (4.38)	52	13.16 (3.77)	49	11.42 (3.29)	50	11.95 (3.91)	151

The instrument used to measure social distance was adopted from the Stigma in Global Context—Mental Health Study (SGC–MHS) and the General Social Survey (GSS) (Bogardus 1959; Martin et al. 2000; Pescosolido et al. 2013; Phillips 1963; Smith et al. 2015). The SGC–MHS/GSS instrument has been used on tens of thousands of respondents, and was developed through an international collaboration involving 16 countries. The reliability (Cronbach’s α) for a 6-item social distance scale from the GSS was found in one study to be .87 (Martin et al. 2000); the items, as used in this study, had a Cronbach’s α of .91. Social distance, the most commonly measured aspect of stigma, refers to the reluctance to interact with members of devalued groups. Social distance items ask respondents to indicate their willingness to have someone with a mental disorder as a neighbor, a friend, a co-worker, an in-law, and so on. All items were coded in this study such that more stigmatizing attitudes receive higher scores, and were administered on a 4-point Likert scale. The items used here and their wording are available as Additional file 1.

### Design and analyses

Multiple linear regression analyses were performed on SPSS 19 to examine the relationship between prevalence estimates and social distance. The analyses were performed only on the respondents asked to provide LMR estimates (*n* = 151), who were not informed about LMR at the start of the survey. The candidate predictors selected for the regression were chosen to facilitate comparisons with Von dem Knesebeck et al. Six candidate predictors (gender, age, educational attainment, personal experience with a mental disorder, contact with persons with a mental disorder, estimated LMR) were selected, nearly the same variables used in Von dem Knesebeck et al. (2013), which also asked for respondents’ city of residence and was therefore a 7-factor model. The results for each term (“mental illness,” “mental disorder,” “mental health condition”) were combined to increase the overall numbers in the regression analysis.

Analysis of variance (ANOVA) was used to examine what effect terminology and informing respondents about LMR might have on the stigma scores. This resulted in a 3 (“mental illness,” mental disorder,” or “mental health condition”) × 2 (either “informed” about LMR at the beginning of the survey or “not informed” and instead asked to provide LMR estimates) fully-crossed factorial design.

## Results

### Estimates and stigma scores

Table 3 shows the results of multiple regression analyses performed on a 6-factor model. The estimates for LMR were significantly associated with respondents’ social distance scores. The 6-factor model in this study predicted 21 % of the variance in social distance scores. In contrast, the 7-factor model from Von dem Knesebeck et al. predicted only 3 % of the variance in social distance.

**Table 3:** Predictors of desired social distance from individuals with mental disorders among respondents not informed about prevalence rates (*n* = 151): results of multiple regression analyses

	B	β	*p*
Constant	16.79		0.00
Gender	0.01	0.00	0.98
Age	−0.02	−0.05	0.55
Education	−0.21	−0.04	0.57
Personal experience	−2.10	−0.21	0.01
Contact	−2.82	−0.30	<0.001
Estimated LMR	−0.03	−0.16	0.05

*R* ^*2*^ = .21 (*p* < 0.001)

*Gender* 0 = male, 1 = female, *Education* 1 = up to 11th grade or less, 2 = up to 12th grade or less, 3 = up to 1 year of college or less, 4 = bachelor’s degree or higher, *Personal experience* 0 = no, 1 = yes, *Contact* 0 = no, 1 = yes

As seen in Table 2, social distance scores for the “not informed” groups were very similar to those for the “informed” groups. This suggests that simply informing respondents about high LMR did not lead to a significant reduction in their stigmatizing attitudes.

### Effects of terminology

There was a main effect of terminology on social distance scores, *F*(2296) = 3.15, *p* = 0.044, and Tukey post hoc tests found that those assigned to the term “mental disorder” (*n* = 99) scored higher than those assigned to “mental health condition” (*n* = 104) (*M*_*diff*_ = 1.38, *p* = 0.038), but not “mental illness” (*n* = 99) (*M*_*diff*_ = 0.97, *p* = 0.20). There were no significant interaction effects found for any of the ANOVA comparisons and no changes in the regression model when effects of terminology were controlled.

## Discussion

This study was intended to examine the relationship between estimates of the prevalence of mental disorders and social distance. The results suggest that higher estimates predict lower desire for social distance. The study was also intended to find the specific term (“mental illness,” “mental disorder,” or “mental health condition”) most likely to evoke these stigmatizing beliefs, and produced some evidence that the label “mental disorder” was more stigmatizing.

### Terminology

The findings of greater stigma associated with the label “mental disorder” stand in contrast with the results from Szeto et al. (2013). It is possible that the term “mental disorder” is, in fact, associated with greater stigma, perhaps by emphasizing the otherness of those stigmatized. The negative findings from Szeto et al. could be related to their use of vignettes, which may have lessened the stigmatizing effects of the label. Participants in that study were also undergraduates receiving extra credit for a psychology course who could have been more familiar with the terms (Szeto et al. 2013). Future research is needed to examine the stigma associated with specific terms, and the way such terms are used and applied in the culture at large.

### Estimates and association with stigma

Estimates for LMR were associated with social distance scores. The regression model shows that an increase of 1 % in a respondent’s prediction for the percentage of Americans who meet criteria for a mental disorder at some point in life (LMR) is associated with a 0.03 point decrease in the respondent’s score for desired social distance. Theoretically, increased LMR estimates from 33 to 100 % might be associated with a drop in social distance scores from the respondents’ mean of 12 to 10, though such assumptions risk extrapolation error. The standardized regression coefficients suggest that LMR estimates are 3/4 as predictive of desire for social distance as actually having a mental disorder, and almost half as predictive of desire for social distance as contact, which is reported to be the most effective anti-stigma intervention (Corrigan et al. 2012). Since increasing contact with individuals with mental disorders may be difficult, these results suggest that changing public beliefs in the LMR for any mental disorder may be a viable option for reducing stigma. If sizeable changes in public desire for social distance can be achieved through interventions as apparently simple as changing beliefs in the LMR for mental disorders, then they may become especially attractive to anti-stigma programs.

The positive results in this study and the negative findings in Von dem Knesebeck et al. may be related to their very different focus: all mental disorders in this study, and individual ones in Von dem Knesebeck et al. (2013). Conceptions about all mental disorders combined could be more related to beliefs held by some respondents about the universal nature of mental disorders. The different results could also reflect other methodological differences between the studies, such as telephone versus online format, use of vignettes, and different scales for social distance (Von dem Knesebeck et al. 2013). Differences in selection bias should also be considered. While 86 % of those who started this study completed and were included in the analysis, the response rate in Von dem Knesebeck et al. was only 51 %.

### Implications for anti-stigma programs

These results suggest that anti-stigma interventions aimed at improving public awareness of the LMR for having any mental disorder hold promise. But they must do more than simply show prevalence estimates. An individual’s beliefs in high LMR may be accompanied by a reflective process that results in lower desire for social distance, and thinking that mental disorders are more common might make them seem more normal. Perhaps future programs may be able to reduce stigma by facilitating this reflective process. Such interventions could also be more successful in adolescents, who respond best with anti-stigma interventions that involve education rather than contact (Corrigan et al. 2012). Since MTurk is only available to adults, it cannot be used to study these younger populations.

Anti-stigma interventions that involve education or information are generally believed to be less effective than those involving contact, but the approaches are rarely studied in isolation. Meta-analyses of interventions for public stigma have found only 2 studies supporting the effectiveness of contact with no adjunct (Thornicroft et al. 2015), and only 1 randomized control trial was effective for the “mental illness” label (Griffiths et al. 2014). Education, its relationship with contacts, and possible mediating factors, such as disclosure, all deserve further study.

### Limitations

The study had a number of limitations. The sample size was small. It was not drawn from the population at large and was conducted online via MTurk. MTurk samples do resemble the population, but they differ in some respects. The ability for MTurk workers to browse for tasks by their descriptions and keywords also creates the potential for bias. Yet few workers use keywords to find tasks, and it is the recency of a task that is posted, its length, and compensation rate, which was generous in this study, that generally determine if an MTurk worker will participate (Paolacci and Chandler 2014). Overall, the anonymous online format appears more likely to elicit the honest responses that are needed to measure these associations, without social desirability bias (Henderson et al. 2012).

Informing respondents about the high LMR of mental disorders did not reduce stigma, potentially limiting the significance of the associations. The strength of the associations in this study was also relatively modest. In the regression model, even the most dramatic changes in perceived LMR do not predict more than 2 points’ improvement in desired social distance. Yet even 1 point on the social distance scale used here is enough to make those probably unwilling to work closely on a job with an individual with a mental disorder, now probably willing. As with all research using social distance scales, however, behavioral responses must not be inferred from reported intentions. And social distance scales are only proxy measures for actual discrimination.

Lastly, stigma associated terms with terms such as “mental illness,” “mental disorder,” or “mental health condition,” will have no impact on the community if the terms are not applied to real behaviors and individuals. Only some disorders are identified as “mental illness” when described in vignettes: 88 % for schizophrenia, 69 % for major depressive disorder, 49 % for alcohol dependence, and 44 % for cocaine dependence (Link et al. 1999), but only 11.3 % for social anxiety disorder, 6.8 % for panic disorder, and 6.1 % for generalized anxiety disorder (Coles and Coleman 2010). Yet the stigma of “mental illness” will remain for as long as these labels are applied to such conditions.

## Conclusions

The term “mental disorder” may be associated with relatively higher stigma than terms “mental illness” or “mental health condition.” Individuals who provided higher LMR estimates for having any mental disorder expressed less desire for social distance. But simply informing individuals about the high LMR of mental disorders does not appear to reduce the stigma associated with the conditions. Future research should identify variables mediating the associations between LMR estimates and stigma.

Even with increasing evidence from prospective studies to suggest the almost universal prevalence of mental disorders in the community, psychiatrists continue to debate the possible effects of accepting and disseminating such information to the public (Moffitt et al. 2010). Yet if stigma may in some way be reduced by accepting and promoting these epidemiological reports, why wait? Public beliefs in the LMR for any disorder must not be ignored for their potential to alter and reduce the public stigma of mental disorders.

## Additional file

10.1186/s40064-016-2974-y Survey items. **Table S2.** Percentages of respondents endorsing a stigmatizing response on social distance items in the current study and in the Stigma in Global Context—Mental Health Study (SGC-MHS) and General Social Survey (GSS) depression and schizophrenia vignettes for the US and world public.
