# Supplementary material for: Public perception of the lifetime morbid risk of mental disorders in the United States and associations with public stigma
Source: Springerplus. 2016 Aug 12;5(1):1342. doi: 10.1186/s40064-016-2974-y (PMC4987741; doi:10.1186/s40064-016-2974-y)
Supplement: Supplementary file 1 — 10.1186/s40064-016-2974-y Survey items. Table S2. Percentages of respondents endorsing a stigmatizing response on social distance items in the current study and in the Stigma in Global Context—Mental Health Study (SGC-MHS) and General Social Survey (GSS) depression and schizophrenia vignettes for the US and world public. [file 40064_2016_2974_MOESM1_ESM.docx]

| **Additional file Table S1.** Survey items. | |
| --- | --- |
| Survey items used in the Stigma in Global Context—Mental Health Study (SGC-MHS) and General Social Survey (GSS)^a^ | |
| Item | Would you be definitely willing, probably willing, probably unwilling, or definitely unwilling . . . |
| 1 | To have [NAME]^b^ as a neighbor? |
| 2 | To spend time socializing with [NAME]? |
| 3 | To have [NAME] care for your children or children you know? |
| 4 | To make friends with [NAME]? |
| 5 | To work closely with [NAME] on a job? |
| 6 | To have [NAME] marry someone related to you? |
|  | |
| Survey items used in the current study^c^ | |
| Item | Would you be definitely willing, probably willing, probably unwilling or definitely unwilling^d^ . . . |
| 1 | To have someone with [a mental disorder]^e^ as a neighbor? |
| 2 | To spend time socializing with someone with [a mental disorder]? |
| 3 | To have someone with [a mental disorder] care for your children or children you know? |
| 4 | To make friends with someone with [a mental disorder]? |
| 5 | To work closely with someone with [a mental disorder] on a job? |
| 6 | To have someone with [a mental disorder] marry someone related to you? |
| ^a^ All items from Smith et al (2015)  ^b^ Survey items in the SGS-MHS were preceded by vignettes describing individuals with depression or schizophrenia, who were referred to by name.  ^c^ Adapted from Smith et al (2015)  ^d^ Coding was assigned so that more stigmatizing attitudes received higher scores. Definitely willing = 1, probably willing = 2, probably unwilling = 3, and definitely unwilling = 4.  ^e^ The terms “mental illness,” “mental disorder,” and “mental health condition” were randomly assigned for each survey in the current study. | |

| **Additional file Table S2.** Percentages of respondents endorsing a stigmatizing response on social distance items in the current study and in the Stigma in Global Context—Mental Health Study (SGC-MHS) and General Social Survey (GSS) depression and schizophrenia vignettes for the US and world public^a^ | | | | | | | |
| --- | --- | --- | --- | --- | --- | --- | --- |
|  | Current study, all respondents^b^ |  | Depression  vignette and diagnosis—  US^c^ | Depression  vignette and diagnosis—  World |  | Schizophrenia  vignette and diagnosis—  US | Schizophrenia  vignette and diagnosis—World |
|  | *n* = 302 |  | *n* = 1425 | *n* = 6539 |  | *n* = 1425 | *n* = 6542 |
| Unwilling to make friends | 13.58 |  | 15.07 | 25.78 |  | 25.91 | 36.95 |
| Unwilling to work closely | 13.58 |  | 35.91 | 32.18 |  | 52.03 | 43.47 |
| Unwilling to socialize with | 13.91 |  | 20.85 | 27.28 |  | 37.74 | 39.93 |
| Unwilling to have as neighbor | 15.56 |  | 14.87 | 14.76 |  | 30.77 | 33.12 |
| Unwilling to have as in-law | 30.13 |  | 59.91 | 58.26 |  | 75.71 | 68.43 |
| Unwilling to care for children | 59.93 |  | 87.45 | 77.27 |  | 94.47 | 85.57 |
| ^a^ Data from Pescosolido et al (2013)  ^b^ Respondents in the current study were given only a 2-3 word description of an individual (with either “mental illness,” a “mental disorder,” or a “mental health condition”) who later became the topic of the survey.  ^c^ Respondents in the SGC-MHS and GSS were given a vignette describing an individual with the condition, informed about the diagnosis, and then asked survey questions about the individual. | | | | | | | |
